# A Complex Network of Sigma Factors and sRNA StsR Regulates Stress Responses in *R. sphaeroides*

**DOI:** 10.3390/ijms22147557

**Published:** 2021-07-14

**Authors:** Katrin M. H. Eisenhardt, Bernhardt Remes, Julian Grützner, Daniel-Timon Spanka, Andreas Jäger, Gabriele Klug

**Affiliations:** Institute of Microbiology and Molecular Biology, Justus-Liebig University of Giessen, IFZ, Heinrich-Buff-Ring 26-32, 35392 Giessen, Germany; kmeisenhardt@gmail.com (K.M.H.E.); Bernhard.Remes@gmx.de (B.R.); Julian.Gruetzner@mikro.bio.uni-giessen.de (J.G.); Daniel_Timon.Spanka@mikro.bio.uni-giessen.de (D.-T.S.); Andreas.Jaeger@mikro.bio.uni-giessen.de (A.J.)

**Keywords:** anoxygenic photosynthesis, photooxidative stress, alternative sigma factor, sRNAs, transcriptome, regulatory networks

## Abstract

Adaptation of bacteria to a changing environment is often accompanied by remodeling of the transcriptome. In the facultative phototroph *Rhodobacter sphaeroides* the alternative sigma factors RpoE, RpoHI and RpoHII play an important role in a variety of stress responses, including heat, oxidative stress and nutrient limitation. Photooxidative stress caused by the simultaneous presence of chlorophylls, light and oxygen is a special challenge for phototrophic organisms. Like alternative sigma factors, several non-coding sRNAs have important roles in the defense against photooxidative stress. RNAseq-based transcriptome data pointed to an influence of the stationary phase-induced StsR sRNA on levels of mRNAs and sRNAs with a role in the photooxidative stress response. Furthermore, StsR also affects expression of photosynthesis genes and of genes for regulators of photosynthesis genes. In vivo and in vitro interaction studies revealed that StsR, that is under control of the RpoHI and RpoHII sigma factors, targets *rpoE* mRNA and affects its abundance by altering its stability. RpoE regulates expression of the *rpoHII* gene and, consequently, expression of *stsR*. These data provide new insights into a complex regulatory network of protein regulators and sRNAs involved in defense against photooxidative stress and the regulation of photosynthesis genes.

## 1. Introduction

In their natural environment, most bacteria are exposed to changing conditions that may limit survival and are considered stresses. Molecular mechanisms that allow bacteria to adapt to and to survive such stress situations have been known for decades. Nevertheless, new players in bacterial regulation, such as sRNAs, small proteins, or small signaling molecules, have been identified over the years and new models of regulation have emerged that are far more complex than anticipated. 

**Figure 1:**
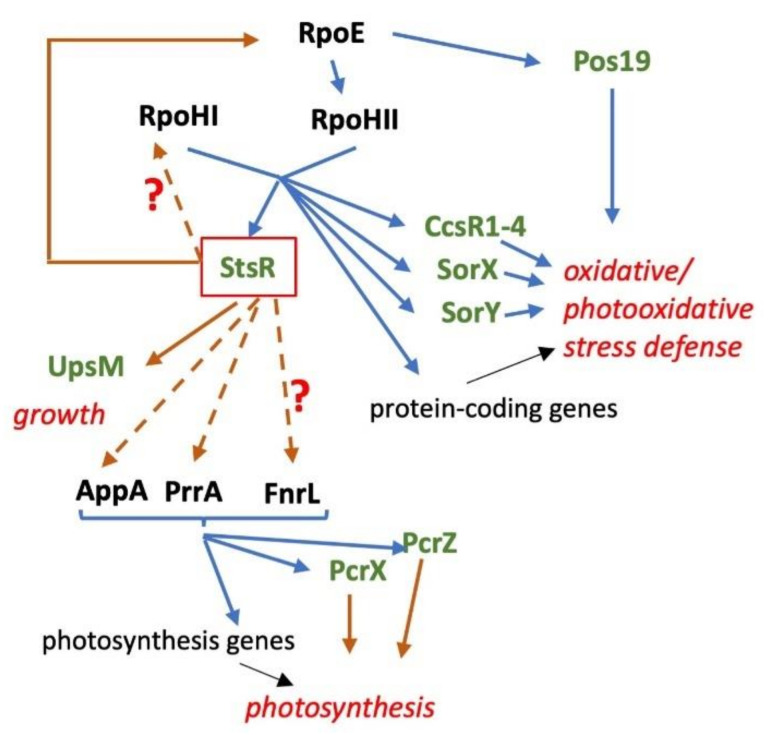
Schematic overview of the regulatory network of sRNAs (green) and proteins (black) affecting the photooxidative stress response and expression of photosynthesis genes. Blue arrows indicate activation, brown arrows repression.

It is widely accepted that adaptation of bacteria to stress conditions occurs mostly at transcriptional level, although in some cases a strong modulation of the proteome is observed that is not accompanied by similar strong changes at the transcriptome level (e.g., adaptation to the stationary phase of *R. sphaeroides* [1]). Transcriptional regulation is often controlled by sigma factors that recognize different promoter sequences and recruit the RNA polymerase to the promoters (reviewed in [2,3,4]). In addition, many DNA binding proteins other than sigma factors are known to influence promoter activities. Several pathways that activate sigma factors or other transcription factors in response to environmental signals have been unraveled in the past (e.g., [5,6,7]). sRNAs make important contributions to post-transcriptional regulation. Among several mechanisms of action, they often influence the translation and/or stability of their target mRNA (e.g., [8,9]). Some sRNAs are controlled by alternative sigma factors and are consequently transcribed in response to external signals (e.g., [10,11]). Here we demonstrate that the sRNA StsR interacts with the mRNA of the RpoE sigma factor in *R. sphaeroides* and thus is part of a regulatory network affecting photooxidative stress defense and the formation of photosynthetic complexes (Figure 1).

*R. sphaeroides* is an Alphaproteobacterium that performs aerobic respiration as long as sufficient oxygen is present. If oxygen tension drops, photosynthetic complexes are assembled into intracytoplasmic membranes and allow the use of light for anoxygenic photosynthesis. If no light is present, anaerobic respiration or fermentation can generate ATP. To avoid photooxidative stress by the production of singlet oxygen, the formation of photosynthetic complexes is controlled by oxygen tension and light. Important factors in this regulation are the two component system proteins PrrA (response regulator) and PrrB (sensor kinase) that senses the electron flow through cbb3 cytochrome oxidase [12,13], the transcriptional repressor PpsR and the antirepressor proteins AppA, that senses oxygen through heme and light through the BLUF domain [14,15,16,17,18], and PpaA that uses cobalamine as a light sensor [19,20]. Furthermore, FnrL is an oxygen-responsive regulator of some photosynthesis genes [21,22,23]. In addition to transcription factors, sRNAs influence the expression of photosynthesis genes by having modulating effects as part of regulatory feed-forward loops [24,25,26,27]. The sRNAs, PcrX and asPcrL, interact with parts of the polycistronic *puf* mRNA that encodes the pigment-binding proteins of the reaction center (RC) and light harvesting (LH) complexes and the assembly factor PufX. They affect the stability of *puf* mRNA segments and, consequently, the stoichiometry of RC/LHI and LHII complexes [26,27]. LHII proteins are encoded by the *puc* mRNAs that are not affected by PrcX or asPcrL. PcrZ negatively affects its targets *puc2A* and *bchN* and thereby counteracts and balances the strong induction of photosynthesis genes upon a drop in oxygen [24,25]. Transcription of these sRNAs is controlled by the same proteins (PrrA, PpsR-AppA, FnrL) as expression of their targets (Figure 1).

The control of photosynthesis gene expression in response to external factors should avoid photooxidative stress. However, sudden changes in oxygen tension and/or light intensity after formation of photosynthetic complexes can take place and lead to photooxidative stress, mostly through the production of the reactive singlet oxygen [28]. A main role in the photooxidative stress response in *R. sphaeroides* was attributed to the alternative sigma factors RpoE, RpoHI and RpoHII [29,30,31]. Under nonstress conditions, RpoE is inactivated by its antisigma factor ChrR [32]. Under oxidative stress, the proteases DegS and RseP promote degradation of ChrR [33]. The proteins RSP_1090 and RSP_1091 promote this process in the presence of singlet oxygen but not in the response to organic peroxides [33]. RpoE targets a relatively small number of genes, including its own gene, the gene for a photolyase, the gene for the sRNA Pos19 and the gene for the RpoHII sigma factor (Figure 1). RpoHII controls a rather large regulon including genes with functions in singlet oxygen quenching, methylglyoxal detoxification, methionine sulfoxide reduction, the GSH-dependent defense and quinone pool retention [30,31]. The RpoHII regulon has considerable overlap with the RpoHI regulon [30,31]. While *rpoHII* mRNA levels show a much stronger increase upon singlet oxygen exposure than after heat shock, *rpoHI* mRNA shows a stronger increase after heat shock [29]. Both RpoH sigma factors also play an important role in the stationary phase and are required for fast outgrowth from the stationary phase [34]. Consensus binding sequences for RpoHI, and RpoHII have been identified [27,28]. 

RpoHI and RpoHII do not only regulate expression of protein-coding genes but also regulate expression of sRNAs with a role in the stress defense in *R. sphaeroides* [15,32,35,36,37] (Figure 1). StsR (formerly RSs0827) was first described as an sRNA induced upon iron starvation [36], and was later found to be the most highly induced RNA in late stationary phase [1,34]. StsR is under control of RpoHI/RpoHII [37] and, therefore, induced by multiple stress factors such as heat and oxidative stress. This sRNA was named StsR (sRNA targeting sRNA) due to its binding to the sRNA UpsM. [38]. UpsM is derived from the 5′ UTR of the *dcw* (cell division and cell wall) genes [38], and binding of StsR to UpsM and to the *dcw* 5′ UTR affects *dcw* gene expression (and consequently cell division) in trans and in cis [37]. Here, we demonstrate that StsR also affects several mRNAs for regulators of photosynthesis genes and photosynthesis gene expression, as well as expression of sRNAs with a role in photooxidative stress in the stationary phase; we identify the *rpoE* mRNA as one target of StsR.

## 2. Results

### 2.1. Overview on the Effect of StsR on Expression of Protein-Coding Genes

The *R. sphaeroides* sRNA StsR shows very low abundance in the exponential phase but is highly abundant in the stationary phase [37]. To evaluate the effect of StsR on the transcriptome, we performed RNAseq analysis with wild type cells of *R. sphaeroides* and with a mutant (ΔStsR) lacking the *stsR* gene. For each strain and condition, triplicates were sequenced, each stemming from a mixture of three independent cultures. 

Principal component analysis (PCA) revealed very good reproducibility within the replicates of every group (Figure S1A). Using DESeq2 [39], the transposed count matrix was used to compute the Euclidean sample-to-sample distances and to perform a hierarchical clustering (Figure S1B). A heatmap revealed strong similarities between the samples from both strains, which were taken during the exponential growth phase. In contrast, the transcriptomes of the wild type and the StsR mutant strain differed greatly during the stationary phase. Within these clusters, the three samples belonging to one strain formed distinct subclusters. The growth phase-dependent differences in the cellular RNA composition were visible in volcano plots: Only few transcripts varied between the strains during the exponential phase, but during the stationary phase more than a third of all transcripts were classified as differentially expressed (Figure S1C,D).

We considered all genes as differentially expressed when the log_2_-fold change between the two strains was ≥1.0 or ≤−1.0 and the adjusted *p*-value was ≤0.05 (Supplementary Table S1). Although StsR showed very low abundance in the exponential phase, 26 protein-coding genes showed higher expression in the mutant. Among those were several genes for flagellar synthesis and chemotaxis. Seventeen protein-coding genes showed lower expression in the mutant, including *znuB* and *znuC* (*znuA* missed the cut-off for *p*-value) for a zinc transporter and *pufK*, which is part of the photosynthesis gene cluster. Seventy two hours after inoculation to an OD_660nm_ of 0.2, *Rhodobacter* cells were in the late stationary phase and StsR was highly expressed [37]. Under these conditions 618 protein-coding genes showed higher expression in the mutant strain (Supplementary Table S1). Among those were *bchI* (*bch* genes are required for bacteriochlorophyll synthesis) and *tspO* from the photosynthesis gene cluster. TspO is an outer membrane protein that negatively regulates expression of photosynthesis genes in response to oxygen by controlling the efflux of porphyrin intermediates [40]. The *fnrL* gene for a regulator of photosynthesis genes also showed higher expression in the mutant. Furthermore, the *rpoHI*, *rpoHII, rpoE* genes, and RSP_3095 for another sigma factor, all showed 4.6-5.7 times greater expression in the *stsR* mutant (Table 1).

In the late stationary phase, 762 protein-coding genes showed lower expression in the mutant (Supplementary Table S1). Among them were several *bch* genes, *pufX* that is required for the assembly of the reaction center (RC) and light-harvesting I (LHI) complexes, and *hemZ* and *hemN* (for oxygen-independent coproporphyrinogen III oxidases that are required for synthesis of protoporphyrin IX).

### 2.2. Effects of StsR on Expression of Photosynthesis Genes and of Genes for Regulators of Photosynthesis Genes

**Figure 2:**
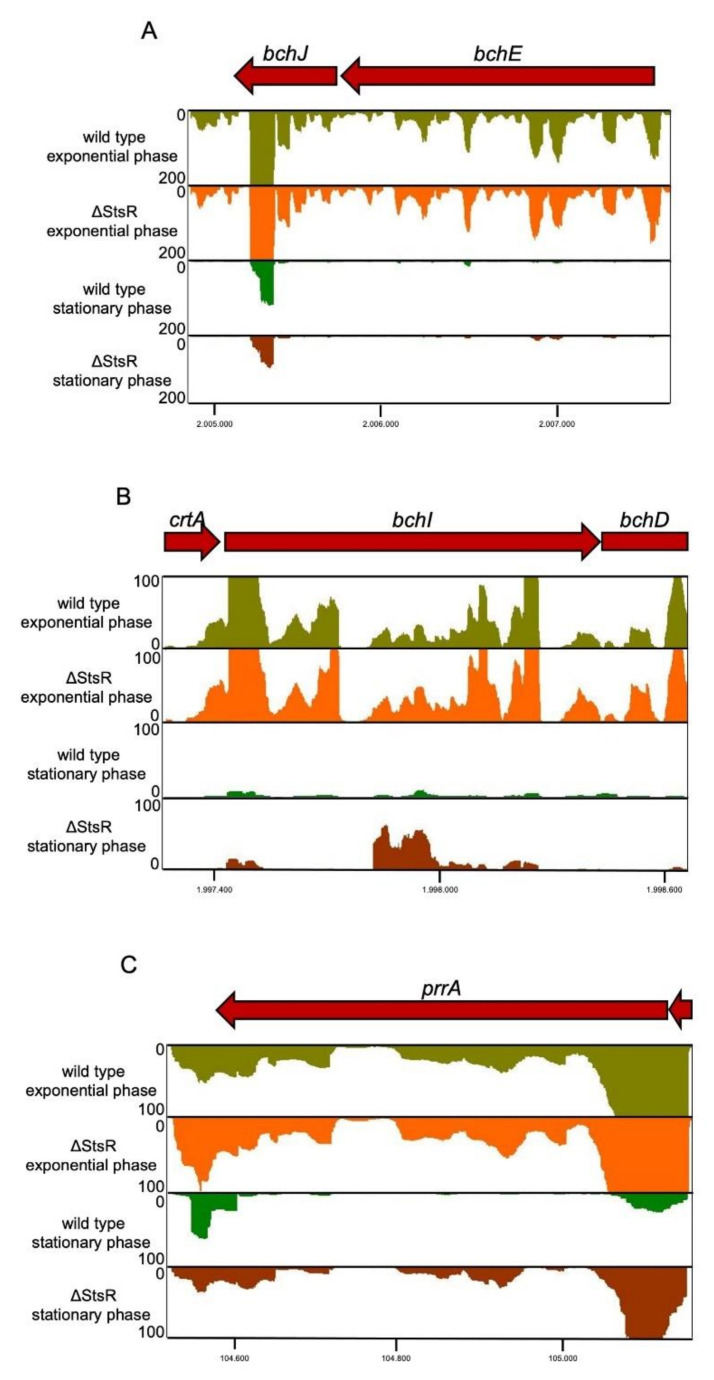
Effect of StsR on expression of selected photosynthesis genes and of the gene for a regulator of photosynthesis genes. Read numbers from RNAseq visualized by the Integrated Genome browser are shown for (**A**) *bchJ* and *bchE* genes, (**B**) the *bchI* gene required for bacteriochlorophyll synthesis and (**C**) the *prrA* gene encoding the response regulator of the PrrB/PrrA two component system. Reads are shown for RNA isolated from the wild type or a mutant lacking StsR (ΔStsR) in the exponential or stationary phase 72 h after inoculation. The read counts within one panel were all normalized to the same scale, as indicated.

Table 1 shows the expression changes for photosynthesis genes, for genes encoding regulators of photosynthesis, and for genes encoding sigma factors involved in stress responses that show log_2_-fold change of ≥0.5 or ≤−0.5 between the two strains in the stationary phase (adj. *p*-value ≤0.05, otherwise numbers are in brackets, and genes with read counts <20 in both strains were excluded). In agreement with the low levels of StsR in the exponential phase, all these genes showed similar expression in the exponential phase in the wild type and mutant. Most photosynthesis genes showed a strong decrease in expression in the stationary phase in the wild type, and an even stronger decrease in the mutant strain. As a result, the mutant showed lower expression in the stationary phase, but transcript levels in both strains were very low compared to exponential growth phase, as shown for *bchJE* in Figure 2A.

A different effect of StsR was observed for *tspO,* and *bchI* (Table 1), which are not part of the same operon (results for *bchI* shown in Figure 2B). Expression levels were more decreased in the stationary phase in the wild type than in the *stsR* mutant, resulting in higher levels of *bchI* and *tspO* mRNAs in the mutant in the stationary phase. The expression pattern of *bchI* differed from that of *crtA*, *bchD* and *bchO*, although all these genes are in the same operon (these genes are not listed in Table 1 due to less than 20 reads in the stationary phase). Higher expression levels in the stationary phase in the mutant were only observed for *bchI.* This strongly suggests that StsR does not affect transcription of the operon but acts at the post-transcriptional level, like most sRNAs.

Transcriptional start sites in the *R. sphaeroides* transcriptome have previously been identified by differential RNAseq [34] (2017; GEO accession number GSE71844). The *tspO* gene is transcribed from a RpoHII-dependent promoter [31]. Since StsR also influences expression of this sigma factor (see below), its effect on *tspO* mRNA levels is likely indirect through altered levels of RpoHII in the mutant.

Altered expression of photosynthesis genes should also affect formation of the photosynthetic apparatus in the *stsR* mutant. Spectral analysis confirmed this assumption: the *stsR* mutant accumulated less photosynthetic complexes than the wild type under phototrophic conditions in the stationary phase (Figure S3).

**Figure 3:**
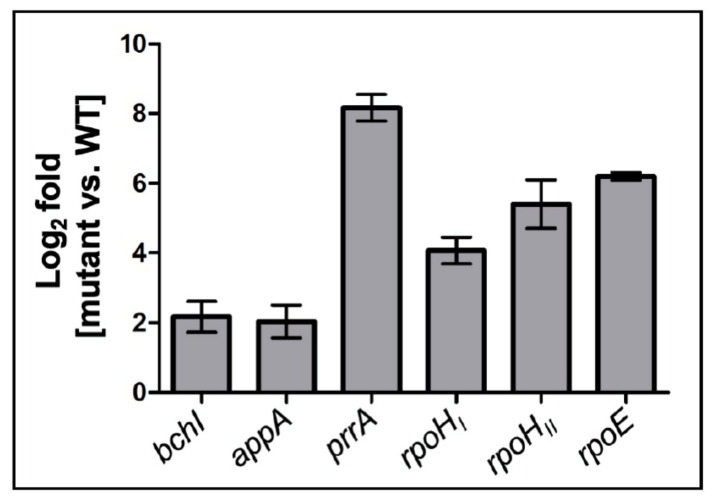
Ratio of expression (log_2_-fold change) of selected genes as determined by real time RT PCR in the ΔStsR mutant compared to the wild type. An in vitro transcript of *sinI* RNA, an external spike-in RNA of known sequence and quantity, was used for normalization.

Our data revealed that StsR also affects expression of some genes for important regulators of photosynthesis genes in the stationary phase (Table 1). This is the case for the *fnrL*, *prrA*, and *appA* genes, which all showed higher expression in the *stsR* mutant in the stationary phase. As observed for photosynthesis genes, expression was similar in the mutant and wild type in the exponential phase and dropped in the stationary phase in the wild type. In contrast to the results for most photosynthesis genes, expression in the stationary phase was higher in the mutant and reached similar levels as in the exponential phase (results for *prrA* shown in Figure 2C). Since the action of StsR on regulatory proteins impacts many other genes and, therefore, is of special importance, we confirmed the RNAseq data by qRT PCR for some selected regulator genes (Figure 3). These data confirmed the higher expression levels of *appA* and *prrA* in the mutant compared to the wild type in the stationary phase. The factors for expression changes are often higher in real time data than in RNAseq due to the high sensitivity of the PCR-based approach. Especially for *prrA,* the change observed by real time RT PCR was much higher. While the DEseq [39] analysis calculates the expression levels based on the read counts for the whole gene, only a small part of the mRNA is amplified in the real time analysis, which can account for such big differences.

### 2.3. Effect of StsR on mRNAs for Alternative Sigma Factors

**Figure 4:**
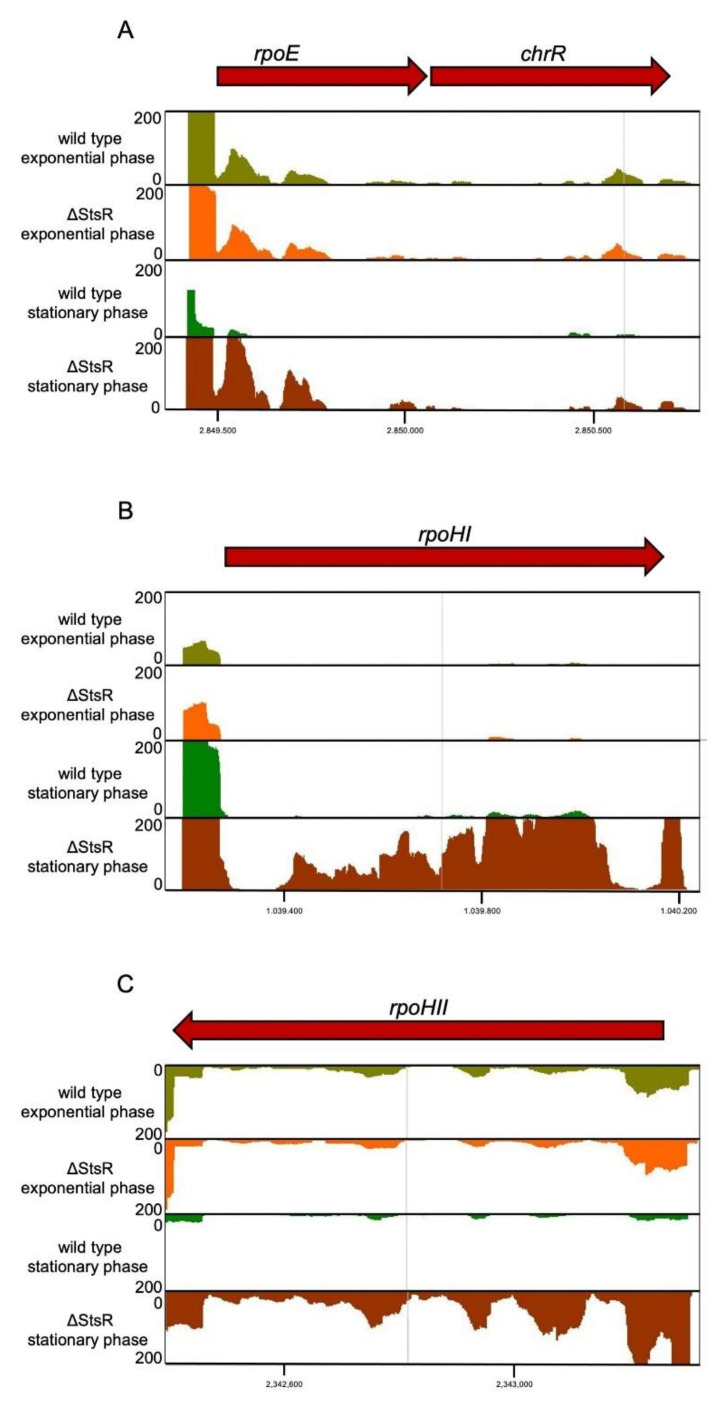
Effect of StsR on expression of selected genes for alternative sigma factors. Normalized read numbers from RNAseq visualized by the Integrated Genome browser are shown for (**A**) *rpoE* and *chrR* genes encoding a sigma factor and its antisigma factor, (**B**) the *rpoHI* gene and (**C**) the *rpoHII* gene encoding alternative sigma factors with an important role in stress responses. Reads are shown for RNA isolated from the wild type or a mutant lacking StsR (ΔStsR) in the exponential or stationary phase 72 h after inoculation. The read counts within one panel were all normalized to the same scale, as indicated.

StsR is strongly expressed in the stationary phase in conditions known to also induce expression of the genes for the alternative sigma factors RpoHI, RpoHII and RSP_3095 in *R. sphaeroides* [1,34,41]. The mRNA levels for all these sigma factors were increased in the stationary phase in the wild type, and even more so in the mutant (e.g., a 150-fold higher level in the mutant in stationary phase than in the exponential phase for RSP_3095) (Table 1). This was not the case for *rpoE* mRNA in the wild type, but in the *stsR* mutant (Figure 4A). Figure 4B,C also shows expression levels for the *rpoHI* and *rpoHII* genes. For all these sigma factor mRNAs, the highest expression was observed in the stationary phase in the mutant, implicating that StsR counteracts high expression in the stationary phase. Real time PCR quantification of *rpoE*, *rpoHI*, and *rpoHII* mRNAs (Figure 3) confirmed their higher levels in the mutant in the stationary phase.

Table 1 also includes the data for RSP_1093, although it did not fulfil the criteria of fold-change and p-value for the difference between the two strains in the stationary phase. RSP_1093 encodes ChrR, the antisigma factor to RpoE [32]. It is noteworthy that the ratio of *chrR* mRNA levels between the two strains did not change to the same extent for *rpoE*, although both genes are transcribed from the same promoter. This strongly indicates additional regulation at the post-transcriptional level. RSP_3095 is cotranscribed with RSP_3094, most likely encoding the antisigma factor to the RSP_3095 protein. The expression pattern for both genes was very similar (Table 1). Expression levels of other alternative sigma factors (4 RpoN sigma factors with a role in nitrogen metabolism [42] and a second RpoE with unknown function) were similar for both strains in the stationary phase. Interestingly, the mRNA level for the house-keeping sigma factor (RpoD) was decreased in the stationary phase in the mutant compared to the wild type (log_2_-fold change: −0.9) (data not shown).

These data demonstrate that StsR affects expression of many genes, especially in the stationary phase. However, these data cannot discriminate between direct effects by binding to target RNAs or indirect effects. The impact of StsR on expression of alternative sigma factors and regulators of photosynthesis genes suggests that many effects on the transcriptome may be indirect.

### 2.4. Effect of StsR on Expression of sRNAs with a Role in Stress Responses or Photosynthesis Gene Expression

Two trans-acting sRNAs (PcrX and PcrZ) are known to affect photosynthesis gene expression [24,26]. As part of incoherent feed-forward loops, they balance the induction of photosynthesis genes upon reduction of oxygen tension. Several sRNAs are induced in response to various stress conditions and were identified as important regulators in the photooxidative stress response of *R. sphaeroides* [43]. The four homologous sRNAs, CcsR1-4, target the mRNA for the FlhR regulator and affect the glutathione pool and the pyruvate dehydrogenase complex [35]. They are cotranscribed with the gene for the small RNA-binding protein CcaF1, that influences maturation and stability of several sRNAs and/or mRNAs [41]. Another sRNA that influences the glutathione pool, Pos19, affects the abundance of numerous mRNAs involved in sulfur-metabolism [44]. SorX targets the mRNA for the subunit of a spermidine transporter [45]. SorY reduces the metabolic flux through the tricarboxylic acid cycle by targeting the mRNA for a malate transporter [46].

**Figure 5:**
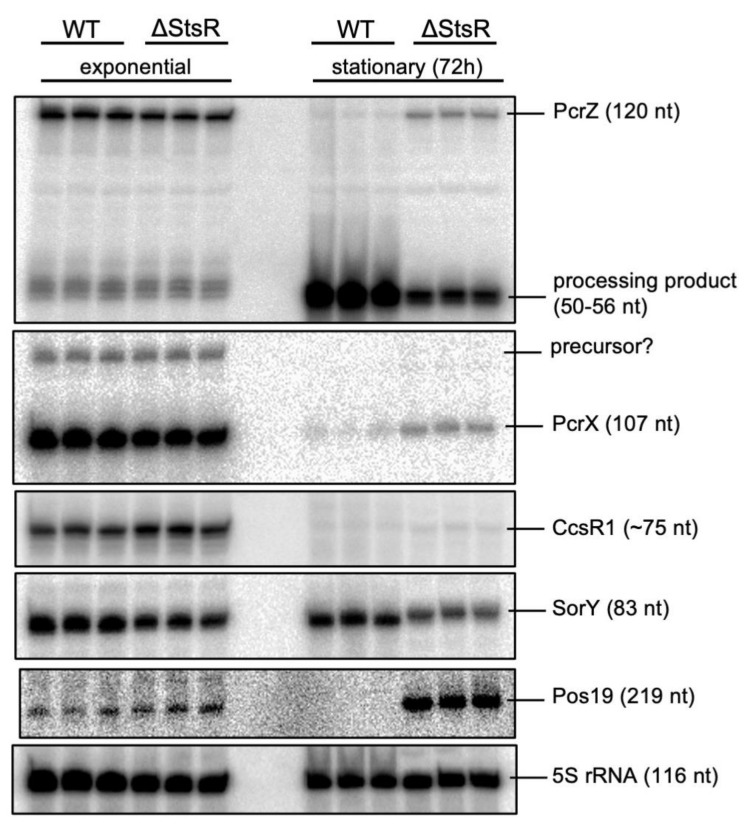
Northern blot of the sRNAs with a role in regulation of photosynthesis genes or in the oxidative stress response in the wild type (WT) and *stsR* mutant in the exponential or stationary phase. For each strain, RNA from three independent cultures was loaded. 8 µg of total RNA were applied to each lane, and 5S rRNA served as loading control. The identical membrane was subsequently hybridized to the specific probes.

These sRNAs are not included in Table 1, since quantification of these short and mostly highly abundant sRNAs by DEseq is often problematic and generates high p-values. We therefore performed northern blots to examine the effect of StsR on the expression levels of these important RNA regulators (Figure 5). For all tested sRNAs, levels in the wild type and mutant were similar in the exponential phase.

PcrZ was previously shown to undergo growth phase-dependent processing: a shorter, stable segment derived from the 5´ end of the PcrZ transcript accumulates in stationary phase [25]. Northern blot analysis revealed that processing of PcrZ was impeded in absence of StsR (Figure 5). It is known that StsR interacts with the UpsM sRNA and promotes its RNase E-dependent cleavage [37]. An interaction between StsR and PcrZ was, however, not predicted by IntaRNA, suggesting a different effect of StsR on PcrZ processing, which may also be indirect.

PcrX and CcsR1-4 are derived from the 3′ UTRs of genes by processing of a precursor transcript. In both cases RNase E has an important role in maturation of these sRNAs [23,33,42]. The amount of PcrX was clearly decreased in the stationary phase, and a stronger decrease was observed for the wild type. StsR had no strong effect on CcsR1 levels, which were significantly lower in both strains in the stationary phase. No processing events are involved in the generation of Pos19 and SorY that are directly transcribed from their genes, and do not undergo further processing [23,34]. Pos19 levels were strongly increased in the mutant but were not detected in the wild type in the stationary phase. SorY had slightly lower levels in the mutant than in the wild type in both growth phases.

Our data demonstrate that StsR can have very different effects on the abundance of individual sRNAs. This effect may also be indirect, mediated by regulatory proteins or other sRNAs. Considering the important functions of the tested sRNAs in regulation, StsR indirectly affects the targets of PcrZ, PcrX, and Pos19 (Figure 1).

### 2.5. StsR Targets rpoE mRNA and Affects Its Stability

**Figure 6:**
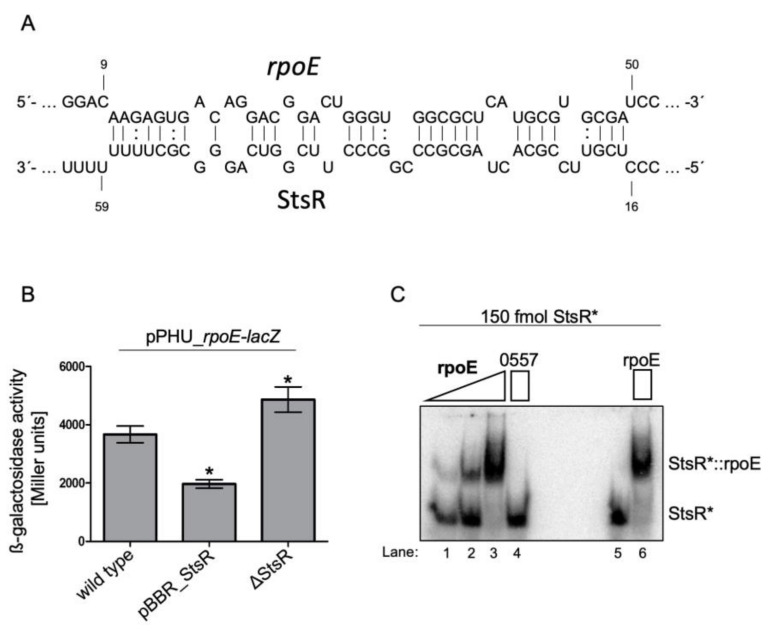
StsR interacts with the *rpoE* mRNA. (**A**) Seed region for the interaction between *rpoE* and StsR as predicted by the IntaRNA tool. (**B**) *lacZ-*based in vivo reporter assay. All strains contain a plasmid with a *rpoE-lacZ* fusion. While over-expression of StsR reduces *rpoE-lacZ* activity, lack of StsR leads to higher expression. (**C**) Gel retardation assay showing the interaction of *rpoE* and StsR in vitro. Radio-labelled StsR (150 fmol) in vitro transcript was incubated with increasing amounts of a 150 nt *rpoE* in vitro transcript (150–15,000 fmol lanes 1–3). A negative control StsR was incubated together with a 100-fold molar excess of an RSP_0557 [23] in vitro transcript (lane 4). As further controls, the StsR transcript was loaded alone (lane 5) or after de and renaturation together with a 100-fold molar excess of the *rpoE* transcript. * *p* < 0.5.

In order to get an idea on putative targets of StsR, we applied IntaRNA [47], a bioinformatic tool for the prediction of RNA-RNA interaction. RpoE mRNA was suggested to be a target of StsR, and an energy value of −18 kJ was calculated for the interaction (Figure 6A; the numbering for *rpoE* mRNA gives the position of nucleotides in relation to the translational start (GUG), numbering for StsR refers to the nucleotide position within the 72 nt long StsR). To verify this interaction in vivo, we compared activity of a *rpoE-lacZ* fusion in the wild type and in the mutant strain. A fragment from position −101 to +111 in relation to the start codon of *rpoE* (not including the promoter of *rpoE*) was cloned into pPHU4352 [24]. In the resulting plasmid (pPHU_1092) the *rpoE* sequence was transcribed from the 16S promoter and translationally fused to *lacZ*. As seen in Figure 6B, introduction of a second plasmid that overexpresses StsR (pBBR_StsR) led to reduced ß-galactosidase activity, while monitoring reporter gene activity in ΔStsR resulted in increased ß-galactosidase activity. This strongly supports the view that StsR reduces expression of RpoE, which is in agreement with the RNAseq data shown in Figure 4A.

To further validate these results, we tested in vitro interaction of the radio-labelled 72 nt StsR and a 153 nt in vitro transcript spanning positions −19 to +134 relative to the *rpoE* translational start (the transcriptional start site for RpoE is at −96 relative to the translational start). As shown in Figure 6C, addition of increasing amounts of the *rpoE* transcript (150–15,000 fmol) resulted in retardation of the radiolabeled StsR (150 fmol), providing further support for direct interaction between these two RNAs.

**Figure 7:**
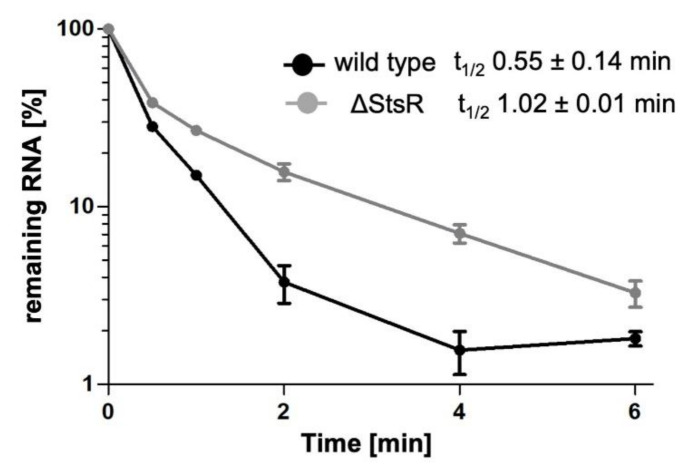
StsR decreases the half-life of *rpoE* mRNA. Rifampicin was added to cultures in the exponential growth phase to inhibit initiation of transcription. RNA was isolated at several time points and *rpoE* mRNA levels were quantified by real time RT-PCR and plotted against time. The values represent the average from three independent cultures, and the standard deviation is indicated. Lack of StsR increased the *rpoE* half-life about two-fold.

Most sRNAs affect translation of their target RNAs by binding close to the translational start, or influence the stability of the target mRNA, or both (reviewed in [8]). The reporter assay shown in Figure 6B cannot discriminate between these mechanisms, since both lead to reduced ß-galactosidase activity. Binding of the sRNA can either stabilize the target by protecting single-stranded regions from cleavage by RNases attacking single stranded regions (e.g., RNase E) or can promote degradation by generating targets for double strand-specific RNases (e.g., RNase III) [8]. Many sRNAs, among them StsR, are associated with the RNA chaperon Hfq [48] that can stabilize the sRNA-mRNA interaction, but can also recruit RNase E and promote destabilization of the target mRNA [49]. As shown in Figure 6A, the seed region for the interaction between StsR and *rpoE* mRNA (purple) is close to the translational start site, starting 10 nt downstream of the GTG. In this region, and just downstream of the interaction site, several RNase E cleavage sites were mapped [50]. To test whether StsR pairing influences *rpoE* mRNA stability, we determined the half-life of *rpoE* mRNA in the wild type and in the *stsR* mutant. Cultures were grown to late exponential phase and rifampicin was added to stop further initiation of transcription. At short intervals after addition of rifampicin, samples were collected for RNA isolation and *rpoE* mRNA was quantified by real time RT PCR. Figure 7 shows that in the strain lacking StsR *rpoE*, the half-life was about doubled compared to the wild type (1.0 min versus 0.55 min). These data strongly suggest that StsR reduces the half-life of *rpoE* mRNA and subsequently its level.

## 3. Discussion

Initiation of transcription is a major check point of regulation of prokaryotes in adapting to their environment, and mechanisms of transcriptional regulation have been studied for decades. Many important protein regulators and regulatory DNA elements have been identified and characterized. Today, the involvement of RNA regulators in adaptation is well recognized, and different mechanisms of this regulation, mostly acting on post-transcriptional levels, have been unraveled [8,9]. Special challenges for facultative phototrophic bacteria are to regulate the formation of the photosynthetic apparatus in order to avoid photooxidative stress and, if this is not possible, to defend against photooxidative stress. This is achieved by a complex network consisting of proteins that regulate transcription, and of sRNAs acting at the post-transcriptional level (Figure 1). The alternative sigma factors RpoE, RpoHI and RpoHII not only control transcription of genes for proteins with a function in stress responses, but also transcription of the sRNAs Pos19, CcsR1-4, SorX and SorY with an important contribution to these responses.

This work attributes a central role to StsR in this network. RpoHI and RpoHII increase transcription of the *stsR* gene in response to stress, while StsR destabilizes *rpoE* mRNA. Thus, RpoE, RpoHII, and StsR form a negative feed-back loop consisting of protein regulators and sRNA. Such regulatory loops have been reported for other bacteria (e.g., [51,52,53,54]). Regulation in the RpoE-RpoHII-StsR loop is based on different mechanisms. RpoE increases transcription of the *rpoHII* gene, while RpoHII increases transcription of *stsR*. The negative effect of StsR on *rpoE* mRNA levels is due, at least in part, to faster turn-over in the presence of StsR. We cannot exclude an additional effect on translation and, consequently, on RpoE protein levels. Since lack of StsR stabilizes the *rpoE* transcript, protection against RNase E cleavage by base pairing of StsR can be excluded. Instead, StsR promotes decay of *rpoE* mRNA. We have recently shown that base pairing of StsR to the sRNA UpsM promotes its cleavage by RNase E. This is due to a structural change of UpsM upon binding of StsR that gives access for RNase E to a previously double-stranded region [37]. A similar mechanism may apply to the effect of StsR on *rpoE* stability.

Our data reveal that *rpoHI* mRNA levels are also increased in the *stsR* mutant (Figure 3 and Figure 4B). The *rpoHI* promoter is not under control of RpoE [31] and no information is available with regard to its activation by stress. Direct interaction of StsR and *rpoHI* was not supported by IntaRNA prediction.

By affecting levels of *rpoE* and *rpoHII* mRNAs, StsR indirectly affects levels of genes that are part of the RpoE and RpoHII regulons, such as the sRNAs Pos19, CcsR1-4, SorX, and SorY (Figure 1). Considering the different expression patterns of these sRNAs, it is obvious that the effect of StsR cannot only be mediated via RpoHII. Lack of StsR increased *rpoE* and *rpoHII* mRNA levels, as seen in Figure 3 and Figure 4. Higher levels of RpoE and RpoHII should increase expression of genes that are controlled by these proteins, which is in agreement with increased levels of Pos19, SorX and SorY in the *stsR* mutant (Figure 5). However, levels of CcsR, PcrZ and PcrX were decreased in the *stsR* mutant (Figure 5). The CcsR1-4 RNAs are transcribed together with an upstream gene, *ccaF1* (RSP_6037). CcaF1 was recently identified as a small RNA-binding protein involved in RNA maturation and turn-over. Increased amounts of CcaF1 interfere with the maturation of the CcsR RNAs from the *ccaF1*-CcsR1-4 precursor transcript and reduce the CcsR1 half-life [41]. These effects can account for reduced CcsR levels even when the *ccaF1*-CcsR promoter is activated.

PcrZ and PcrX are not under control of RpoE or RpoHI/HII but are regulated by PrrA, AppA and FnrL [24,26]. Lack of StsR results in increased levels of mRNAs for these regulators in the stationary phase, and in reduced levels of PcrZ and PcrX. PrrA and FnrL are activators of gene expression, AppA is an antirepressor of PpsR and, consequently, indirectly activates gene expression. We cannot exclude that StsR affects PcrZ and PcrX levels through other mechanisms.

StsR also affects photosynthesis gene expression. Most photosynthesis genes showed lower expression in the mutant in the stationary phase than in the wild type, while the opposite effect of StsR was observed for *bchI* and *tspO*. Regardless of the different expression levels of most photosynthesis genes in the two strains (up to four-fold), these differences may not be of physiological relevance, since expression in the stationary phase was very low in both strains. The effect of StsR on *bchI*, *prrA*, *appA*, and *fnrL* are likely to have larger impact, since only in the absence of StsR were significant amounts of the mRNAs observed. Thus, StsR has an important role in reducing expression of these genes in the stationary phase.

At present, it is not possible to explain the effect of StsR on expression of the individual genes. Although the function of AppA/PpsR, PrrB/PrrA and FnrL have been addressed in numerous studies (rev. [55]), we are far from understanding this complex regulatory network for photosynthesis gene expression. Elucidation of the underlying mechanisms is not straightforward: mutation or overexpression of one gene will at the same time affect other regulators, and the regulatory loop may compensate for the effects caused by the altered level of a single component of the loop. In vitro experiments with only two components may give some more insights into the mechanisms of regulation. For example, identifying direct targets of StsR, and investigating its effects on its targets as shown here for RpoE, may provide helpful information. We do not know, at present, whether some mRNAs for photosynthesis genes, for protein regulators, or for PcrZ and PcrX, are directly targeted by StsR. Nevertheless, in vitro experiments cannot completely mimic the in vivo situation and, in most cases, cannot include the effect of changing environments. Indeed, the complexity of the regulatory network for regulation of the photooxidative stress response, and for photosynthesis gene expression, is even greater, as outlined in Figure 1. StsR can bind the RNA chaperone Hfq [37] that affects many cellular processes. Deletion of Hfq in *R. sphaeroides* has pleiotropic effects, including reduced pigmentation and altered photooxidative stress response. More than 70% of the Hfq-bound sRNAs are affected by singlet oxygen [56]. In the exponential phase, about 60% of the RNA-bound Hfq protein is bound to UpsM (formerly RSs0682) [56]. UpsM is highly abundant in the exponential phase but strongly decreases in stationary phase due to the action of StsR that promotes degradation of UpsM by RNase E. StsR reaches high levels in the stationary phase similar to UpsM in the exponential phase, and also binds Hfq [37]. The sRNAs CcsR, SorY, SorY and Pos19 are known to interact with Hfq, and the function of PcrX is affected by Hfq [26,47,57]. Competition among targets over Hfq binding plays an important factor in regulation (e.g., [57,58,59,60,61,62,63]).

StsR has strong effects on the expression of other genes, mostly in the stationary phase, which is rarely included in studies analyzing bacterial gene expression. In natural habitats, however, bacteria are in the stationary phase for most of the time, so that regulation at this state should not be ignored.

Taken together, our study demonstrates that regulation of photosynthesis genes and of the oxidative stress response in *R. sphaeroides* is far more complex than was anticipated in the past. Complex regulatory loops complicate the elucidation of the role of individual components in regulation. Most likely, it will take a lot more studies to know all components of these complex networks, to understand their interaction and the process of adaptation to different growth conditions.

## 4. Materials and Methods

### 4.1. Bacterial Strains, Plasmids and Growth Conditions

The wild type *R. sphaeroides* 2.4.1 [64] was used for this study. Construction of the mutant strain lacking StsR (2.4.1 Δ*S*tsR), and of plasmid pBBR_StsR for overexpression of StsR is described in [37]. For cultivation of *R. sphaeroides* strains at 32 °C, malate minimal-salt medium was used [65]. Cultures were grown under microaerobic growth conditions, with a dissolved oxygen concentration of about 25–30 µM within the exponential phase. Erlenmeyer flasks containing 80% culture by volume were shaken at 140 rpm. For phototrophic cultivation, the strains were incubated in sealed Metplat flasks filled to the top and illuminated with 60 Wm^−2^ of white light. When necessary, kanamycin (25 µg mL^−1^), tetracycline (2 µg mL^−1^) or spectinomycin (10 µg mL^−1^) was added to liquid and solid growth media (1.6% agar).

### 4.2. Construction of the rpoE-lacZ Fusion

For the *rpoE*-*lacZ* translational fusion, a 218 nt fragment of the *rpoE* gene was amplified with the primer pair rpoE_f and rpoE_r (Table S2). The fragment was subcloned into the pDrive cloning vector (Qiagen, Hilden, Germany) and the *rpoE* sequence was excised by XbaI and HindII and ligated into the corresponding sites of the pPHU4352 [24]. The resulting reporter plasmid pPHU_1092 (Tc^r^) carried the translational *rpoE-lacZ* fusion under control of the 16S rRNA promoter (RSP_4352 promoter) and was transferred into *R. sphaeroides* strains by conjugation as described in [66].

### 4.3. β-Galactosidase Activity Measurements

For measuring ß-galactosidase activity, strains carrying the plasmid with the translational *rpoE-lacZ* fusion under control of the 16S rRNA promoter were incubated in biological triplicates at 32 °C under microaerobic conditions. ß-galactosidase activity was measured by the hydrolysis of O-nitrophenyl-β-D-galactopyranoside (ONPG) (Serva, Heidelberg, Germany) and expressed as Miller Units. Strains were grown until they reached an OD660 of 0.6. Cells were harvested, and the assay was performed as described in Klug et al. [67].

### 4.4. RNA Isolation

*R. sphaeroides* cultures from three independent starter cultures were inoculated separately and grown in triplicate to OD_660nm_ 0.5. For northern blot analysis, quantitative real-time RT-PCR and RNAseq analysis, RNA was isolated using the hot phenol method [68]. Afterwards, the RNA was precipitated with 1/10× vol. 3 M sodium acetate pH 4.5 and 2.5× vol. 96% ethanol.

### 4.5. Northern Blot Analysis

For Northern Blot analysis 10% polyacrylamide/urea gels were used to fractionate 8 µg total RNA, as described earlier [69]. Oligodeoxynucleotides were used for end-labelling with [γ-^32^P]-ATP (SRP-30; Hartmann Analytic, Braunschweig, Germany) by T4 polynucleotide kinase (#EK0031, Fermentas, Ontario, Canada). A low stringency Church buffer was used for hybridization. Membranes were washed in 5× SCC buffer + 0.1% SDS. After exposure on phosphoimaging screens (Bio-Rad), images were analyzed by 1D-Quantity One software (Bio-Rad, Feldkirchen, Germany). Oligonucleotides used for hybridization are listed in Table S2.

### 4.6. Quantitative Real-Time RT PCR

For qRT-PCR, total RNA was isolated using peqGOLD TriFast™ (VWR) as described by the manufacturer. Afterwards the RNA was treated with TURBO DNA-free™ Kit (Ambion/ ThermoFisher Scientific, Waltham, MA, USA) to remove DNA contaminations. For qRT-PCR, the Brilliant III Ultra-Fast SYBR^®^ Green QPCR Master Mix was used for reverse transcription and PCR, as described in the manufacturer’s manual. Each 10 µL reaction mixture contained 5 µL Master Mix (supplied), 0.1 µL DTT (100 mM, supplied), 0.5 µL RiboBlock solution (supplied), 0.4 µL water, 1 µl of each primer (10 pmol/L) and 2 µL DNA-free RNA (20 ng/µL). The reactions were performed in a spectrofluorometric thermal cycler (Biorad, Feldkirchen, Germany) and were visualized with BioRad CFX Manager 3.0. For all qRT-PCR experiments, means and standard deviations of biological triplicates were calculated, each performed in technical duplicates. For all primers, a no template-control was included. The expression of the target mRNAs in the strain of interest was calculated relative to the respective control strain and an in vitro transcript of *sinI* RNA, an external spike-in RNA of known sequence and quantity, was used for normalization [70]. Primers are listed in Table S1.

### 4.7. Gel Retardation Assay

For gel retardation assays, RNA was transcribed in vitro using T7 Polymerase (NEB, Massachusetts, USA) and PCR products with a T7 promoter region at the 5′ ends as the template. The assays were carried out with 150 fmol radio-labelled in vitro transcript and various molar ratios of nonlabelled in vitro transcripts in a final volume of 8 µL. RNAs were denatured separately for 1 min at 95 °C and renatured by cooling for 2 min on ice and for 5 min at 32 °C. After these de and renaturing steps, the radio-labelled and nonlabelled RNAs were mixed and 4 µL of 5× structure buffer (25 mM MgCl_2_ and 300 mM KCl) were added for a final volume of 20 µL. For complex formation, the samples were incubated for 30 min at 32 °C. Afterwards, the reactions were mixed with 5 µL of loading dye (50% glycerol, 0.5× TBE, 0.2% bromophenol blue) and loaded onto a 6% nondenaturing polyacrylamide gel containing 0.5× TBE. Gels were pre-run at 100 V for 60 min at 4 °C before loading. Electrophoresis was performed at 4 °C by applying 200 V for 4 h. Gels were dried, exposed on phosphoimaging screens (Bio-Rad, Feldkirchen, Germany) and analyzed using 1D-Quantity One software (Bio-Rad, Feldkirchen, Germany).

### 4.8. RNAseq Analysis and Evaluation

RNA isolation, library preparation and bioinformatic analysis were performed as previously described [34,50]. DESeq2 (version 1.16.1; [39]) was applied for quantitative comparison of the data, and the p-value (Benjamini–Hochberg correction) was calculated. The data are deposited in GEO under the accession number GSE175997. Coverage plots in wiggle format representing the number of aligned reads per nucleotide were generated based on the aligned reads and visualized in the Integrated Genome Browser [71]. The raw coverage values of the graphs were normalized to the total number of reads that could be aligned for the respective library and multiplied by the minimum number of mapped reads of all libraries.

### 4.9. Half-Life Determination of the mRNA

RNA samples were prepared at different time points after addition of rifampicin to the cultures as described in RNA isolation and quantification. Half-lives were calculated based on real time RT-PCR with 20 ng of total RNA for both the target gene and for the standard gene *rpoZ.*

## Figures and Tables

**Table 1:** log_2_-fold changes in read counts determined by RNAseq within a strain between different growth phases or between wild type (WT) and the *stsR* mutant in the same growth phase, as calculated by DEseq2 [39]. Brackets indicate that the adjusted *p*-value is >0.05. Growth curves for the two strains are shown in Figure S2 and the time points of sampling are indicated.

Gene		Log_2_-Fold WT Station./ Expon. Phase	Log_2_-Fold Mutant Station./ Expon. Phase	Log_2_-Fold Mutant/WT Expon. Phase	Log_2_-Fold Mutant/WT Station. Phase
Photosynth. genes					
*pufX*	RSP_0255	−2.54	−3.79	(−0.14)	−1.26
*pufM*	RSP_0256	−2.65	−3.23	(−0.43)	−0.58
*bchY*	RSP_0261	−2.02	−3.57	(0.04)	−1.56
*bchX*	RSP_0262	−2.24	−3.14	0.25	−0.90
*tspO*	RSP_0269	−1.67	(−0.34)	(−0.03)	1.33
*bchI*	RSP_0273	−2.35	−1.26	0.28	1.10
*bchJ*	RSP_0280	−2.40	−4.00	−0.69	−1.67
*bchE*	RSP_0281	−1.66	−2.51	(−0.01)	−0.85
*bchH*	RSP_0287	−1.41	−2.86	(−0.07)	−1.00
*bchL*	RSP_0288	−1.18	−2.03	(0.15)	−0.85
*hemN*	RSP_0317	1.09	−2.70	(0.29)	−1.90
*hemZ*	RSP_0699	−1.12	−3.02	(0.05)	−1.95
Genes for regulatory proteins					
*fnrL*	RSP_0698	−0.82	1.00	(0.03)	1.80
*prrA*	RSP_1518	−1.32	−0.52	0.49	0.86
*appA*	RSP_1565	−0.80	−0.06	(−0.04)	0.70
Genes for alternative sigma factors/anti-sigma factors					
*rpoHII*	RSP_0601	1.10	3.33	(−0.04)	2.22
*rpoE*	RSP_1092	(−0.15)	2.89	(−0.15)	2.42
*chrR*	RSP_1093	−1.85	2.23	(−0.23)	(0.15)
*rpoHI*	RSP_2410	3.69	6.20	(0.06)	2.52
	RSP_3095	5.04	7.23	(0.12)	2.19
	RSP_3094	5.32	6.61	(0.32)	1.50

## Data Availability

The RNAseq data are available in the NCBI gene expression omnibus repository (GEO accession number GSE71844).

## Supplementary Materials

The following are available online at https://www.mdpi.com/article/10.3390/ijms22147557/s1, Figures S1–S3 and Tables S1 and S2.
