# Supplementary material for: A Complex Network of Sigma Factors and sRNA StsR Regulates Stress Responses in R. sphaeroides
Source: Int J Mol Sci. 2021 Jul 14;22(14):7557. doi: 10.3390/ijms22147557 (PMC8307010; doi:10.3390/ijms22147557)
Supplement: Supplementary file 1 [file ijms-22-07557-s001.zip › Supplementary Material.pdf]

Supplementary Material

Table S1. Expression levels based on DEseq2 analysis for genes with a log_2-fold change ≥1.0 or ≤−1.0 between the wild type and ΔStsR and an adjusted *p*-value ≤ 0.05: see separate Excel-file.

Table S2. Oligodeoxynucleotide sequences for cloning, real-time RT-PCR and northern blot.

**Oligodeoxynucleotide Cloning**	**Sequence 5′–3′**
rpoE_f	TCTAGAAGGACGTTAAGATCACGGC
rpoE_r	AAGCTTCACCTTCGGCGCAAAG
T7_StsR_f	TAATACGACTCACTATAGCCGTTCTACCTT CACTGTC
T7_StsR_r	AAAAAAAAGCGCCCTGAC
T7_rpoE_f	TAATACGACTCACTATAGGGCAAGGGGA
T7_rpoE_r	CTGCCGGATTTCATCAGGAAG
**Real-tTime RT-PCR**	
*rpoZ*_A	ATCGCGGAAGAGACCCAGAG
*rpoZ*_B	GAGCAGCGCCATCTGATCCT
*rpoE*_A	GTCTGGCAGAAGGCTCAT
*rpoE*_B	GTTCTCCTGCTGCATCTC
$rpoH_{II}$_A	GCCGATGAACGACCTGAT
$rpoH_{II}$_B	AAGAACAGCGCCTTCTGG
$rpoH_{I}$_A	GATCGCCAAGGATCT
$rpoH_{I}$_B	CTGGTCGCTGTCTTCA
*prrA*_A	AACTCGGGGCCGACAGG
*prrA*_B	GCCTGGGCGATCGCCT
*appA*_A	ACGGCCCTGATATTCAGGTTCG
*appA*_B	TGTTGCATCCTTCGCCCCTTAC
*bchI*_A	GGGCGCGCTCGACATCGA
*bchI*_B	ACTGCGCCACGTCGAGGA
**Northern Blot**	
p_CcsR1	CGTCGCCGCTGCTGCTACAGGTC
p_PcrZ	GCAGTCGCCGGATACTCGTTACC
p_SorX	CCGGGAAGCGCGAGAGAAG
p_SorY	ATGAAGCGGACGAGAGAACCCTC
p_PcrX	AAGGGAACCGGGCTGTGGTGTC
p_5S	CTTGAGACGCAGTACCATTG

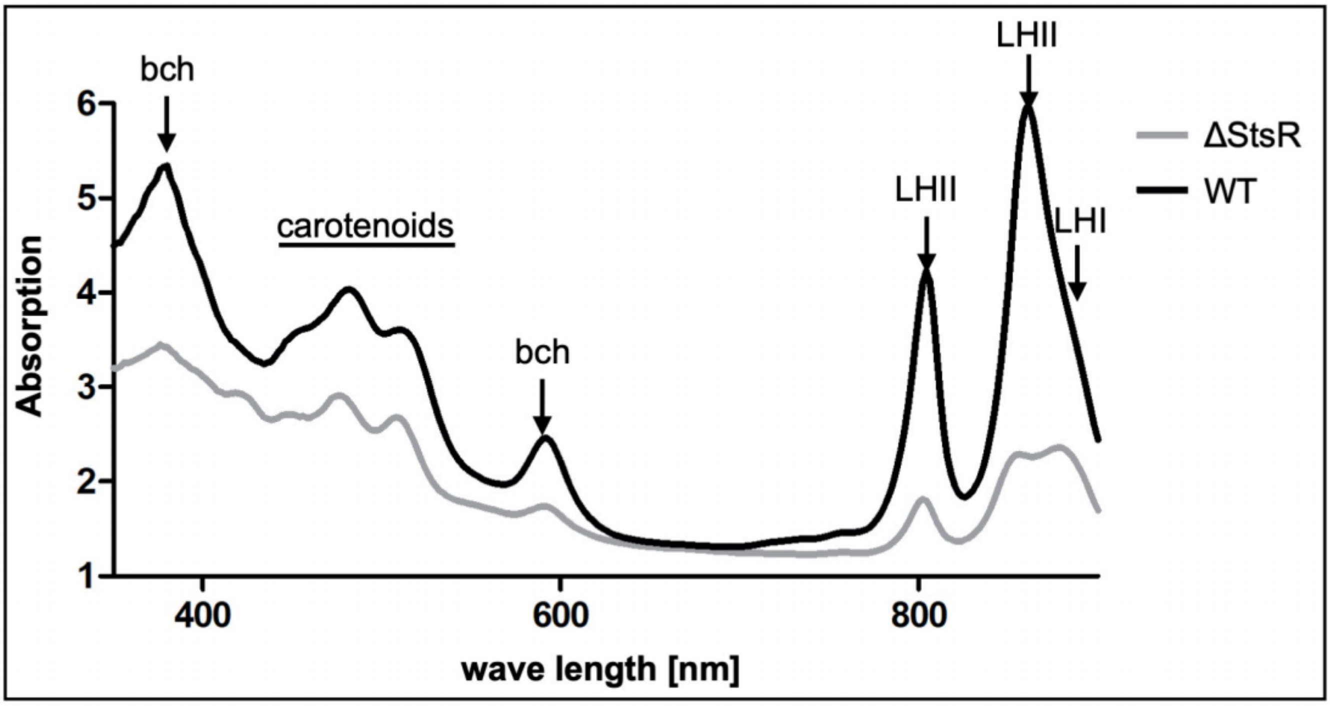

Figure S1. Absorption spectra of wild type (black) and DStsR (grey) grown under phototrophic conditions to an OD660 of 1.5. The absorption peak at 800 nm is mostly caused by LHII complexes, the peak at 860 nm by LHI and LHII (LHI absorbance: 855 nm, LHI absorbance: 875 nm). Peaks at 375 nm and 590 nm stem from absorbance of bacteriochlorophyll independently to its protein association. Absorbances between 430 nm and 520 nm stem from carotenoids.

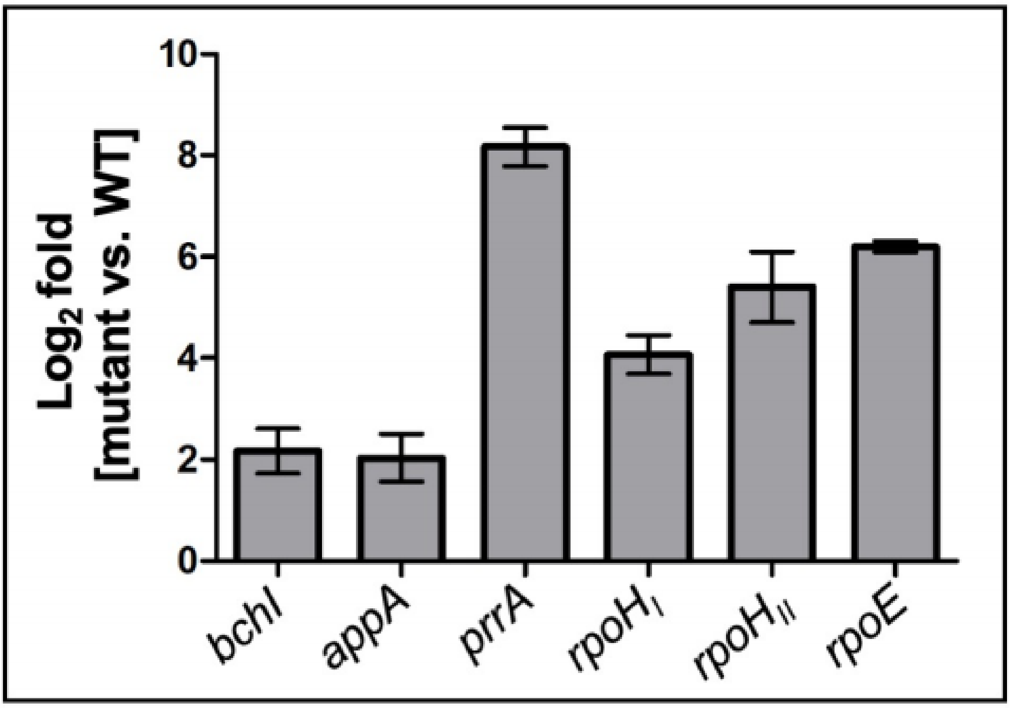

Figure S2. Ratio of expression (log2-fold change) of selected genes as determined by real time RT PCR in the DStsR mutant compared to the wild type. An in vitro transcript of sinI RNA, an external spike-in RNA of known sequence and quantity, was used for normalization.

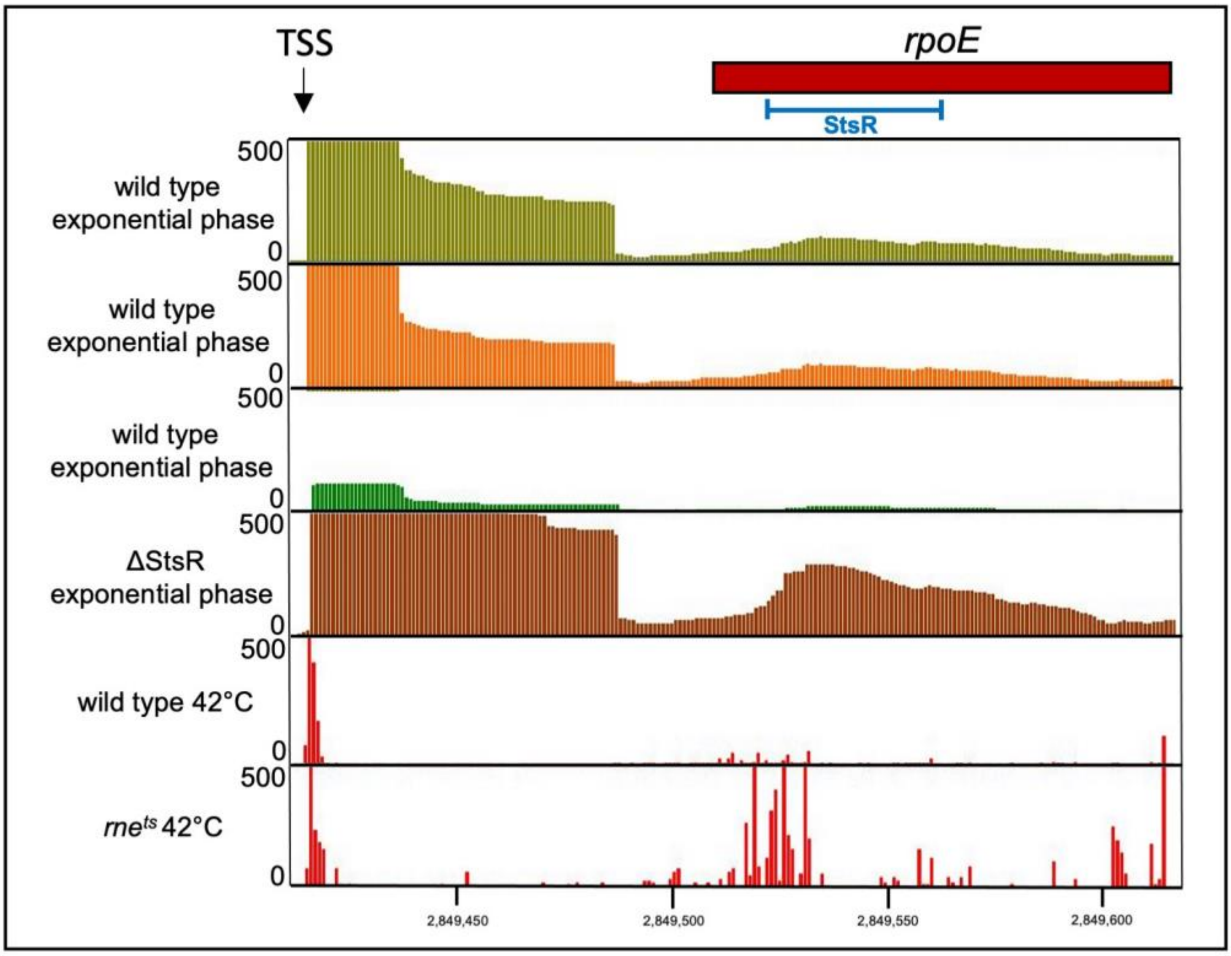

Figure S3. RNase E cleavage sites within the rpoE mRNA. Normalized read counts from RNAseq. visualized by the Integrated Genome browser are shown. The 4 upper panels show total reads, while the two lower panels only show mapped 5′ends. 5′ends that are more abundant in the wild type at 42 °C than in the mutant with a temperature sensitive RNase E (rnets) indicate RNase E dependent cleavage. The transcriptional start site (TSS) is indicated and the blue bar marks the rpoE region that interacts to StsR.
